# Mycotoxin Determination in Peaches and Peach Products with a Modified QuEChERS Extraction Procedure Coupled with UPLC-MS/MS Analysis

**DOI:** 10.3390/foods12173216

**Published:** 2023-08-26

**Authors:** Hong Xie, Yinping Li, Jiaxing Li, Yinglong Chen, Jing Li, Lixue Kuang, Syed Asim Shah Bacha, Tiejun Zhang, Yuehui Chao

**Affiliations:** 1 School of Grassland Science, Beijing Forestry University, Beijing 100083, China; 2 Research Institute of Pomology, Chinese Academy of Agricultural Sciences, Xingcheng 125100, China; 3 The UWA Institute of Agriculture, UWA School of Agriculture and Environment, The University of Western Australia, Perth, WA 6001, Australia

**Keywords:** peach, mycotoxin, QuEChERS, UPLC-MS/MS

## Abstract

Peaches are the most significant temperate fruit crop worldwide. However, peach fruits are susceptible to fungal and mycotoxin contamination. Consequently, monitoring the residual levels of multiple mycotoxins in peaches and related products is essential. In this study, a novel method based on QuEChERS extraction, followed by ultra-high-performance liquid chromatography–tandem mass spectrometry (UPLC-MS/MS) detection, was developed for analyzing 14 mycotoxins in peaches and peach products from China. Matrix-matched calibrations were employed to accurately quantify the mycotoxins and compensate for matrix effects. Recoveries for the target analytes ranged from 84.6% to 117.6%, with intra-day and inter-day precision below 20%. The limits of quantification were 2 or 5 μg/L for the 14 mycotoxins. This method was utilized to detect the presence of target mycotoxins in 109 fresh peaches, 100 diseased peaches, and 89 peach products from China. Six mycotoxins were identified in the rotten parts of the diseased peaches, with concentrations ranging from 5.2 to 1664.3 µg/kg. In the remaining parts of the diseased peach samples, only two toxins, alternariol (AOH) and alternariol monomethyl ether (AME), were quantified at levels of 15.3 µg/kg and 15.5 µg/kg, respectively. No mycotoxins were detected in fresh peaches. For peach products, all contamination levels were below the quantitative limits and significantly lower than the maximum legal limits established for the products.

## 1. Introduction

Mycotoxins are toxic secondary metabolites with low molecular weight produced by toxigenic fungi, and are commonly found in a variety of cereals, fruits, and vegetables [1,2,3,4]. Mycotoxins in agricultural products are primarily produced by molds of genera *Aspergillus*, *Penicillium*, *Fusarium*, *Claviceps*, and *Alternaria* [5,6,7,8,9,10]. To date, 400 types of mycotoxins have been reported, including aflatoxins (AFs), fumonisins (FBs), trichothecenes, zearalenone (ZEA), ochratoxin (OTA), and patulin, which are the most prominent and widely studied [9,11]. Mycotoxins are garnering increasing attention for their toxicity, teratogenicity, carcinogenesis, mutagenicity, nephrotoxicity, estrogenic activity, immunotoxicity, and skin toxicity [12]. The presence of mycotoxins in agricultural commodities can cause adverse health effects for consumers, both humans and animals [9,13]. According to the International Agency for Research on Cancer (IARC), aflatoxin is classified in Group 1 as carcinogenic to humans, while AFB1, AFB2, and OTA are included in Group 2B, possibly carcinogenic to humans [14,15]. Regarding certain toxins, such as citrinin (Group 3), patulin (Group 3), ZEA (Group 3), T2 (Group 3), and *Alternaria* toxins, it should be noted that limited or inadequate evidence exists of their carcinogenicity to humans, but strong exposure to these toxins may still pose a potential carcinogenic risk [16,17,18].

Peaches are the fourth-largest deciduous fruit crop, are widely cultivated globally, and are considered one of the most commercially valuable *Rosaceae* species [19,20]. Peaches are favored by consumers for their unique flavor, rich aroma, and abundant nutrients, generating CNY 460 million in annual revenue (https://www.fao.org/faostat/ (accessed on 8 July 2023)). Peaches are susceptible to fungal colonization, whether from pathogenic fungi in the field or mold fungi during the postharvest and transportation stages [21]. Peach fruit is highly vulnerable to the disease known as brown rot caused by *Monilinia* spp.; peach rot caused by *Rhizopus stolonifer*; peach gray mold caused by *Botrytis cinerea*; and stone fruits caused by *Cladosporium* spp., *Penicillium* spp., *Aspergillus* spp., and *Alternaria* spp. [21,22]. Contaminated peaches designated for processing, especially those in concentrated fruit juice, may pose health risks to consumers. Consequently, many countries and organizations have established the maximum level of mycotoxins in cereals, fruits, and nuts to protect consumers’ health [23,24,25]. The European Commission, the United States, Canada, and China have determined maximum levels of patulin and OTA in fruit juice as 50 μg/kg and 2 μg/kg, respectively [26,27]. The European Commission also stipulates that the maximum levels of AFB1 and total aflatoxins (sum of AFB1, AFB2, AFG1, and AFG2) in dried fruit and processed products are 5 μg/kg and 10 μg/kg, respectively [27]. Furthermore, the occurrence of mycotoxins is monitored by different regulatory bodies worldwide to assure food safety [26,28].

Accurate mycotoxin detection in fruits and derived products are vital for food safety. Numerous analytical methods have been employed to control mycotoxins in fruits, such as enzyme-linked immunosorbent assay (ELISA), thin-layer chromatography (TLC), gas chromatography–mass spectrometry (GC-MS), high-performance liquid chromatography (HPLC), and ultra-high-pressure liquid chromatography–tandem mass spectrometry (UPLC-MS/MS) [29,30,31,32]. ELISA and TLC can be used for qualitative and semi-quantitative detection of mycotoxins [9]. GC-MS and LC-fluorescence detections require derivatization procedures for mycotoxin samples, and many mycotoxins lack fluorescence characteristics [33]. In recent years, UPLC-MS/MS has emerged as the most powerful analytical tool for qualitative and quantitative analysis of mycotoxins due to its superior sensitivity, specificity, and efficiency [30,34]. High demands are placed on sample pretreatment for mass spectrometric instruments, requiring efficient extraction of target analytes while minimizing matrix interference [35]. Since the introduction of the QuEChERS method by Anastassiades in 2003, its distinctive advantages—being Quick, Easy, Cheap, Effective, Reliable, and Safe—have led to its widespread application in the detection of contaminants such as pesticides, mycotoxins, and antibiotics [10,36]. China is the world’s largest producer of peaches, accounting for approximately 50% of global production [37]. It is also the largest consumer of peaches, which are enjoyed as fresh fruit, dried fruit, juices, wines, jams, and sauces. There is little information about the presence of mycotoxins in the peaches and peach products of China. The purpose of this study is to develop and validate a QuEChERS and UPLC-MS/MS method to determine 14 mycotoxins in peaches and four types of peach products. Additionally, the developed analytical method was applied to investigate the occurrence and contamination of multi-mycotoxins in various peaches and peach products in China. This research provides, for the first time, information on multiple mycotoxin occurrence in peach and related products from China.

## 2. Materials and Methods

### 2.1. Reagents and Chemicals

HPLC-grade acetonitrile, methanol, formic acid, and acetic acid were obtained from Thermo Fisher Scientific Inc. (Fair Lawn, NJ, USA). Citric acid (purity ≥ 99%) and anhydrous magnesium sulfate (MgSO_4_, purity ≥ 99%) were acquired from the Institute of Fine Chemicals Zinke (Tianjin, China). NaCl (Purity ≥ 99%) was purchased from Tianjin Kemio Chemical Reagent Co., Ltd. (Tianjin, China). Graphitized carbon black (GCB) and C18 were sourced from Shanghai Kang standard chemical Co., Ltd. (Shanghai, China), and primary secondary amine (PSA) was obtained from Tianjin Zehao Technology Co., Ltd. (Tianjin, China).

Mycotoxin standards AFB1, AFB2, AFG1, AFG2, alternariol (AOH), alternariol monomethyl ether (AME), tentoxin (TEN), Beauvericin (BEA), ZEA, OTA, ochratoxin B (OTB), and three enniatins (ENA, ENA1, ENB1) were purchased from Pribolab Pte. Ltd. (Purity ≥ 98%, Qingdao, China). The stock standard solutions were prepared individually by dissolving each standard in acetonitrile to a final concentration of 100 μg/mL. All standard solutions were maintained in storage under freezing conditions.. A series of working solutions were prepared freshly with the solution of acetonitrile and stored at 4 °C. The ultrapure water used was produced by Milli-Q Synthesis (Millipore, Bedford, MA, USA).

### 2.2. Sampling

**Figure 1:**
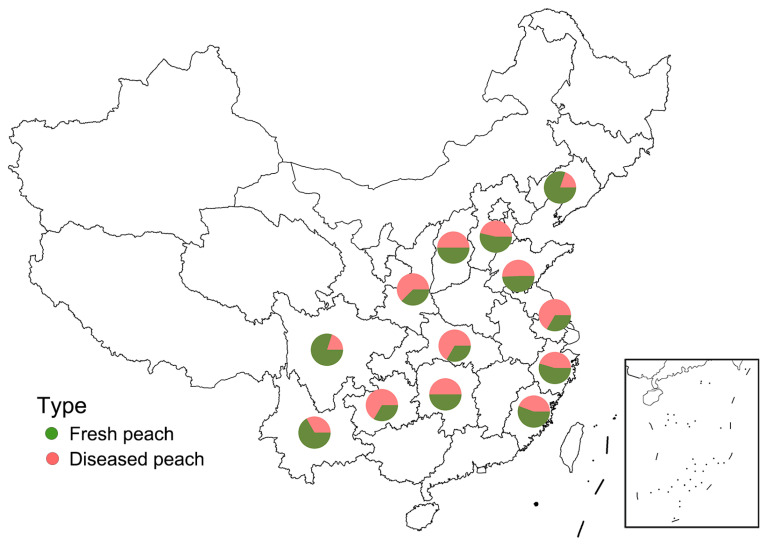
Maps of China showing the peach sampling locations. Pie chart indicates the percentage of fresh and diseased peaches in a province.

The samples included fresh peaches, diseased peaches, and four types of peach products. Firstly, we collected fresh peach samples from 109 locations in Beijing and 13 provinces (Figures S1 and S2 and Table S1). Each individual location was considered as one sample. The sampling and mixing methods of peaches refer to Li, Zhang, Nie, Bacha, Yan, and Gao (2020) [38]. The mixed fresh peaches were frozen in a refrigerator. Simultaneously, diseased peaches were collected from these orchards, with 3–5 diseased peaches from each orchard. A total of 100 diseased peaches were collected (Figure 1 and Table S2). Each diseased peach was divided into two parts: the diseased part (Part A) and the remaining part (Part B). Each part was ground separately and kept in a freezer. For peach products, 89 items were purchased from local markets and online supermarkets, including 14 peach juices, 28 peach wines, 17 dried peaches, and 30 canned peaches. Dried peaches and canned peaches were homogenized with a tissue blender and stored under chilled conditions. Other peach products were stored according to the instructions.

### 2.3. Sample Preparation and Extraction Procedure

Peach, dried peach, and canned peaches were weighed (10 g) and placed into 50 mL polytetrafluoroethylene (PTFE) centrifugal tubes. Peach wine and peach juice samples were accurately measured (10 mL) and transferred into 50 mL PTFE tubes.

To enhance the extraction efficiency of mycotoxins from peaches and their products, four types of extraction solutions were used in this experiment, including acetonitrile, acetonitrile containing 10 mM formic acid, acetonitrile containing 10 mM acetic acid, and acetonitrile containing 10 mM citric acid. The optimization of the extract was initially carried out on the peach samples. Mycotoxins were extracted from the peaches with these four extracts separately. To each sample, 20 mL extraction solution was added and vortexed for 2 min (IKA VORTEX 3). Next, 4 g of anhydrous MgSO_4_ and 1 g of NaCl were added, and the samples were shaken for 2 min (IKA VORTEX 3). After centrifugation at 9000 rpm for 5 min (CF16RX II, Hitachi Koki, Tokyo, Japan), 1.5 mL of the supernatant was transferred to a 2 mL tube with an amount of sorbent. The comparison of different sorbents was carried out using 50 mg PSA/C18/GCB, 100 mg PSA/C18/GCB, and 150 mg PSA/C18/GCB at the same mycotoxin concentration levels (10 μg/mL). Each tube was vortexed vigorously for 1 min before centrifugation at 12,000 rpm for 5 min. Finally, the supernatant (0.9 mL) was filtered through 0.22 μm PTFE filters (FINE Scientific Ltd., Toronto, ON, Canada) prior to HPLC-MS/MS analysis.

### 2.4. Chromatography and Mass Spectrometric Conditions

Chromatographic separation was performed by using a Waters Acquity UPLC system coupled to a Waters Xevo TQ triple quadruple mass spectrometer (Waters Corp., Milford, CT, USA). UPLC separation was achieved on an ACQUITY UPLC™ BEH C18 column (100 mm × 2.1 mm, i.d., 1.7 μm particle size, Waters), with the column maintained at 40 °C.

To obtain a better peak shape and sensitivity of mycotoxins, we optimized a random combination of four aqueous phases A and two organic phases B. There are four types of aqueous phase A, including 0.1% formic acid, 5 mM ammonium acetate, 5 mM ammonium acetate (0.1% formic acid), and 0.1% ammonia. Mobile phase B is methanol and acetonitrile. The gradient program used as follows: 0–2 min, 10% A; 2–4 min, 90% A; 4–4.01 min, 90%–10% A; 4.01–5.5 min, 10% A. All target compounds were eluted within 5.5 min. The gradient elution has a flow rate of 0.4 mL/min, and the injection volume was set at 3 μL.

The mass spectrometer was operated in both positive and native ESI mode with multiple reaction monitoring (MRM) mode. The typical parameters were capillary voltage of 0.5 kV, ion source temperature of 150 °C, desolvation temperature of 400 °C, a desolvation gas (N2) flow of 800 L/h, and a nebulizer gas (N2) flow of 50 L/h. The optimized MS/MS conditions for the 14 mycotoxins were listed in Table 1. The obtained data was processed by MassLynx™ 4.1 software (Waters Corp., Milford, CT, USA).

### 2.5. Method Validation

The UPLC-MS/MS method for 14 mycotoxins in peaches and their derived products was validated in accordance with European Commission Regulation and guideline [39]. The main validation parameters included linearity, limit of detection, limit of quantification (LOQ), recovery, matrix effect, precision, and accuracy. Linearity was obtained using seven matrix-matched calibration points for all target analytes. The LOD was considered as the lowest concentration of the 14 analysts in five matrices that could be detected under the stated test conditions [40]. According to the guidelines of SANTE/11813/2017, LOQ was defined as the minimum spiked level with sufficient recovery and precision [41].

RSD and recovery were used to evaluate the precision and accuracy of the method for each mycotoxin. Recoveries were determined by spiking six duplicate blank matrix samples at spiked levels of 5, 10, 50, and 100 μg/mL. For intra-day precision, six parallel extractions for each matrix on the same day were analyzed. Four spiked levels on three successive days were analyzed for inter-day precision. The matrix effect was calculated by comparing the slope of the calibration curve in the matrix-matching standards (peach, peach juice, peach wine, dried peach, and canned peach) and the solvent standard according to the following equation: Matrix effect (%) = (slope of matrix-matching standard/slope of solvent standard − 1) × 100%.

### 2.6. Statistical Analysis

The data was subjected to analysis using both Microsoft EXCEL 2021 and GraphPad Prism 9.5.0 software. A comparative evaluation of the effects of distinct extractants and detergents on the recovery of targeted mycotoxins was conducted through the application of a two-way ANOVA and subsequent Tukey test. Significance levels were determined based on *p*-values below 0.05.

## 3. Results and Discussion

### 3.1. UPLC-MS/MS Optimization

A highly sensitive MRM method was used to selectively detect and quantify mycotoxins in peaches and their products. MRM parameters were rapidly optimized by directly injecting individual standard solutions. To select the optical form of precursor, the ionization of analytes in positive- and negative-ionization modes in the ESI source was studied. For each analyte, the qualitative and quantitative changes were monitored (Table 1 and Figure S3), which complies with the recommendations of Directive 2002/657/EC [39].

In establishing the UPLC-MS/MS method, adjusting and optimizing the composition of the mobile phase significantly affected on the formation of protonated ions and the chromatographic separation process [10]. In this experiment, we found that 14 mycotoxins exhibited higher ionization efficiency and better peak shape with a combination of 5 mM ammonium acetate containing 0.1% formic acid and methanol among the random combination of four types of phases A (0.1% formic acid, 5 mM ammonium acetate, 5 mM ammonium acetate containing 0.1% formic acid, and 0.1% ammonia) and two types of phase B (methanol and acetonitrile) (Figure S3). In addition, the gradient elution time was also optimized. Under this mobile phase, we detected that the separation of all target mycotoxins was completed within 5 min. 

### 3.2. Optimization of Extraction and Purification Procedure

The sample preparation method is crucial for the analysis of complex matrix samples. In recent years, the QuEChERS method has been the most popular sample preparation method for mycotoxin residue analysis in various matrices. Therefore, the extraction and purification steps were optimized to improve the sample preparation efficiency. Peach was selected as the model matrix, and the method was applied to other matrices after optimization.

#### 3.2.1. Selection of Extractants

**Figure 2:**
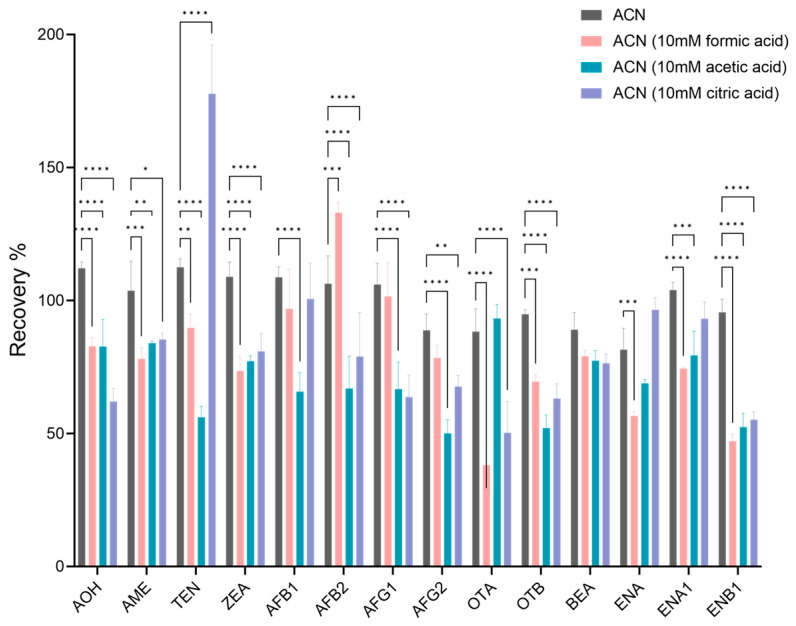
Effect of different extractants on the recoveries of targeted mycotoxins. All data analyzed by two-way ANOVA and the Tukey test (* *p* < 0.05; ** *p* < 0.01; *** *p* < 0.001; **** *p* < 0.0001).

The selection of extraction conditions is mainly related to the physical and chemical properties of the target compounds and the properties of the solvents [10]. Acetonitrile with appropriate polarity for most analytes was selected as the extraction solvent [40]. For some mycotoxins sensitive to polarity ranges, it is reported that adding acid to the extractant can help improve the recovery of mycotoxins [10,42]. The effects of acetonitrile, acetonitrile containing 10 mM formic acid, acetonitrile containing 10 mM acetic acid, and acetonitrile containing 10 mM citric acid on the recovery rates of 14 mycotoxins were compared (Figure 2). The results showed that the recoveries of OTA, ENA, and ENB1 were less than 60% and AFB1 was more than 130% when formic acid was contained in acetonitrile. When acetic acid was present in acetonitrile, the recovery rates of OTB, ENB1, and AFG2 were less than 60%. When citric acid was contained in acetonitrile, the recovery rates of OTA and ENB1 were less than 60%, and the recovery rate of TEN was more than 130%. When using pure acetonitrile solution for extraction, the recovery rates of 14 mycotoxins ranged from 81.57 to 102.53%. The additions to acetonitrile did not significantly improve the recoveries of most mycotoxins. The addition of acid in acetonitrile can improve or reduce the recovery rate of specific mycotoxins [10,42]. However, using acetonitrile as the extraction solvent met the required recovery rates of 14 mycotoxins in this study, so acetonitrile was selected as the extraction solvent. Diverging from established extraction protocols that involve sample dilution [43,44], certain methodologies emphasize concentration, particularly when dealing with samples containing mycotoxins that are typically present in low concentrations [30]. In our experimental procedure, we opted for a direct analysis without the use of dilution or concentration steps during extraction, as fungal toxins in peach samples are also found at relatively lower levels.

#### 3.2.2. Selection and Dosage of Detergents

**Figure 3:**
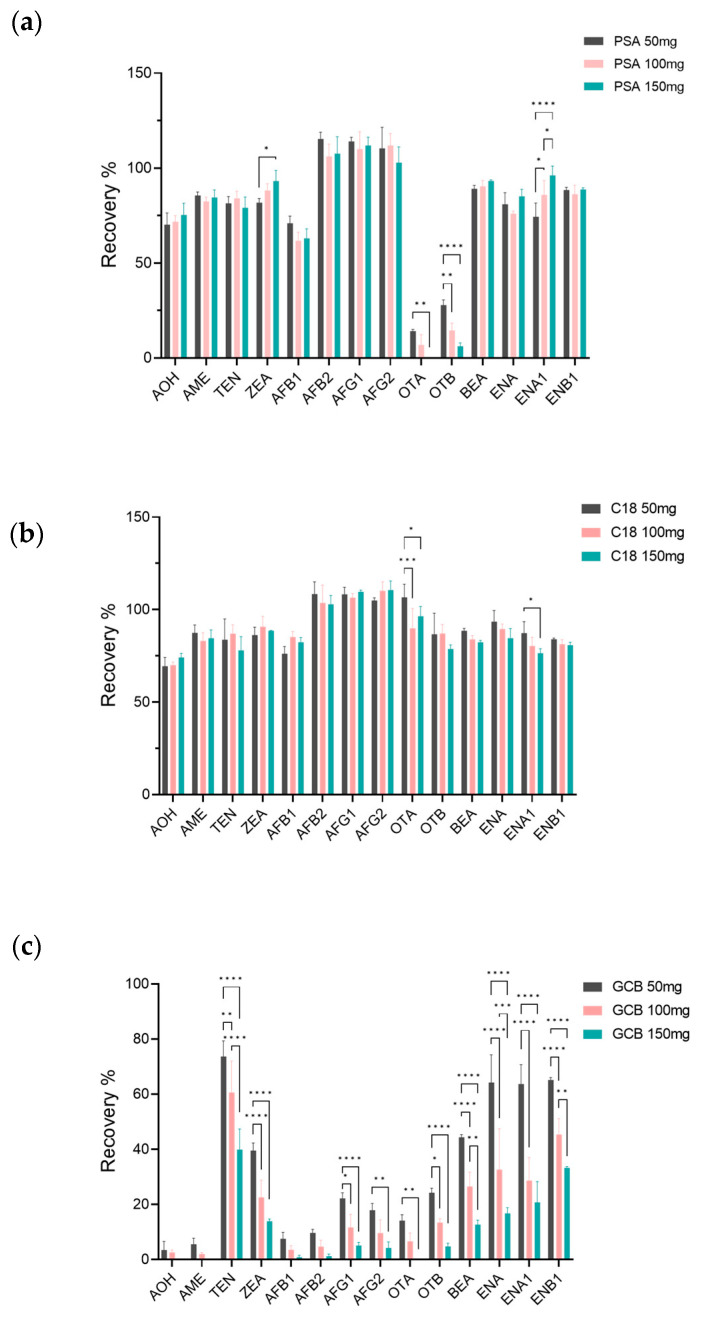
Comparison of recovery of target mycotoxins in peach matrix using varying amounts of PSA, C18, and GCB. (**a**) Distribution of recoveries obtained using 50, 100, and 150 mg of PSA during the clean-up process for mycotoxins in peach matrix; (**b**) distribution of recoveries obtained using 50, 100, and 150 mg of C18 during the clean-up process for mycotoxins in peach matrix; (**c**) distribution of recoveries obtained using 50, 100, and 150 mg of GCB during the clean-up process for mycotoxins in peach matrix. All data was analyzed by two-way ANOVA and the Tukey test (* *p* < 0.05; ** *p* < 0.01; *** *p* < 0.001; **** *p* < 0.0001).

Because of the complex matrix of peach samples and the presence of many compounds [45], chromatographic separation may be affected, enhancing or reducing the ion signal of target analytes [40]. Therefore, a purification step was required prior to UPLC-MS/MS analysis. C18, PSA, and GCB are typical adsorbents used for QuEChERS purification [13,40,46]. PSA sorbent is mainly used to remove polar interferences such as sugars and organic acids from the sample extracts, C18 has been proven effective in removing non-polar interferents, and GCB has been used to remove pigment compounds, such as carotenoids and chlorophyll [13,40,47]. In this study, the purification efficiency of the three adsorbents was studied using the working solution of the standard mixtures, and the recovery rates of the target analytes were evaluated (Figure 3). The recovery results showed that PSA met the required recovery rates except OTA and OTB, and GCB adsorbed most mycotoxins, so the recovery rates did not meet the requirements. The influence of the dosage of C18 (50, 100, and 150 mg) on the recoveries of 14 mycotoxins in the samples was further investigated. The results showed that, as indicated in Figure 3, when 150 mg C18 was added to each milliliter of extract, the recovery rates of 14 mycotoxins ranged from 74.1% to 110.5667%, and the RSDs ranged from 0.33 to 9.48%. Therefore, the recovery and RSD were both satisfactory for all the target analytes when 150 mg C18 was used in each peach sample, and the purification effect was optimal.

### 3.3. Method Validation

In accordance with the guidelines of Commission Regulation No. 519/2014 [48] and Commission Decision 2002/657/EC [39], we assessed the linearity, LOD, LOQ, matrix effect, recovery, accuracy, and precision of the established method using optimized conditions.

#### 3.3.1. Linearity, LOD, and LOQ

Linearity was determined by preparing different calibration curves (working standard solution, peach, peach juice, peach wine, dried peach, and canned peach matrices) within the concentration of 2–1000 μg/L for all the analytes. The calibration curves demonstrated excellent linearity, with high correlation coefficients (R^2^ ≥ 0.99), ranging from 2 to 1000 μg/L (Table 2). The LODs for the 14 mycotoxins in all matrices were estimated to be between 0.001 and 0.5 μg/L. The LOQs for the target compounds ranged from 2 to 5 μg/L.

#### 3.3.2. Matrix Effect

**Figure 4:**
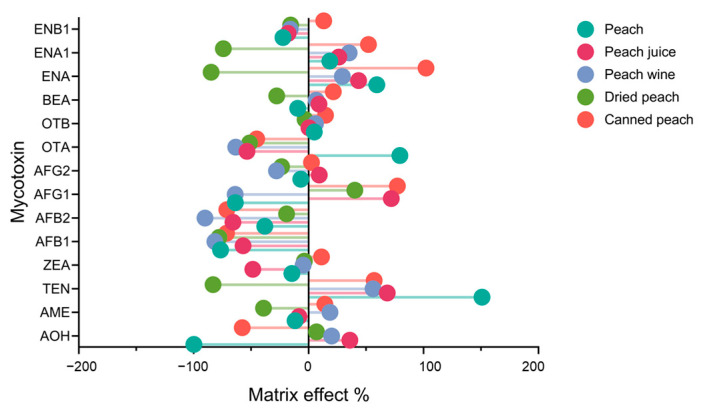
Matrix effect distribution of 14 mycotoxins in peaches and four peach products.

The matrix effect refers to the phenomenon where the co-eluted matrix components reduce the detection sensitivity and accuracy of target analytes in chromatographic separation [30,40,49]. In this study, the matrix effect was calculated by comparing the slope in the matrix with the slope generated by the standard corresponding to the concentration of the matrix standard prepared in the solvent. For all mycotoxins analyzed in the study, matrix effect (%) values between −20% and 20% were considered as low matrix effects; values between −50% and −20% or 20% and 50% were considered as medium matrix effects; values less than −50% or more than 50% were considered as high matrix effects [47]. As shown in Figure 4 and Table 2, ENA, TEN, OTA, AFG1, and AFB1 exhibited medium to high matrix effects in all matrices, while OTB and ENB1 displayed low matrix effects across all matrices. To ensure accurate quantification, a matrix-matched calibration curve was applied to minimize errors and obtain the results closest to the actual results in the samples.

#### 3.3.3. Recovery and Precision

Precision and accuracy studies were conducted by spiking matrix samples at different concentration levels (5, 10, 50, and 100 μg/L) for each analyte. Intra-day precision and accuracy were assessed by injecting six replicates of each concentration level on the same day. Inter-day precision and accuracy were determined by injecting three replicates of each concentration level every day over three days. The validation results of the inter-day and intra-day precision and accuracy are presented in Table 3. The average recoveries of all target compounds ranged from 84.6% to 117.6%. The intra-day precision ranged from 0.7 to 17.4%, while the inter-day precision ranged from 0.5 to 19.1%; both intra-day and intra-day precision values were lower than 20% [48]. The impact of the sample matrix on the analysis could potentially lead to reduced levels of consistency and precision in the assay results [50]. However, this analytical methodology showcases remarkable uniformity across diverse batches. The above results indicated that the method exhibited good accuracy and precision.

### 3.4. Mycotoxin Detection in Peach

The presence of target mycotoxins in fresh peaches and diseased peaches was determined and quantified using the optimized method. The targeted mycotoxins were not detected in any fresh peach samples, consistent with findings in fresh apples and tomatoes [38,51]. All target mycotoxins were detected in diseased peaches, and the types and concentrations of mycotoxins differed between the rotten and remaining parts of the diseased peaches (Part A and Part B). A total of 14 mycotoxins were present in the rotten part of the diseased peaches, with 6 mycotoxins exceeding the LOQ: AOH, AME, TEN, AFB1, OTA, and BEA. The positive rates of these six mycotoxins were from 4.1% to 50.8%, and the mean contamination levels ranged from 5.2 µg/kg to 1664.3 µg/kg (Table 4). Among the mycotoxins with concentration exceeding the LOQ in the rotten part of the diseased peaches (Part A), AOH, AME, and TEN had relatively high detection rates. In the remaining part of the diseased peaches (Part B), only two kinds of mycotoxins, AOH and AME, with mean concentrations of 15.3 µg/kg and 15.5 µg/kg, exceeded the LOQ (Table 4). AOH, AME, and TEN belong to *Alternaria* mycotoxins. AOH and AME have been associated with human esophageal cancer, while TEN is considered to be related to plant chlorosis [38]. AFB1 is the most carcinogenic of aflatoxins and has been linked to hepatocellular carcinoma, growth inhibition, immune system regulation, and malnutrition [52]. In this study, AFB1 was detected in only one rotten part of a diseased peach, with a concentration of 41.9 µg/kg. Exposure to OTA leads to nephrotoxicity, hepatotoxicity, immunotoxicity, and neurotoxicity [52]. The OTA concentration in the rotten parts of four diseased peaches exceeded LOQ, with a mean value of 13.0 µg/kg and median value of 13.0 µg/kg. BEA is a *Fusarium* mycotoxin, and its toxicity has been shown to adversely affect the gastrointestinal tract, immunity, and steroid production [49]. The BEA concentrations in the rotten parts of three diseased peaches exceeded LOQ, with a mean value of 49.5 µg/kg and a median value of 13.7 µg/kg. A remaining part of a diseased peach had a BEA concentration exceeding LOQ, with a concentration of 5.2 µg/kg.

The method was also used to detect target mycotoxins in four peach products (Table 5). There were eight, nine, nine, and eleven mycotoxins detected in peach juices, peach wines, dried peaches, and canned peaches, respectively. BEA had the highest detection rates among the mycotoxins. However, the concentrations of mycotoxins detected in peach products were between the LOD and LOQ. Mycotoxins in peach products likely originated from contaminated raw materials. As mold can rapidly colonize peaches and produce mycotoxins under favorable conditions, it is speculated that mycotoxins in peach products may come from rotten peaches or contamination during the production process. However, the levels of target mycotoxins in peach products in this study were quite low, which may not pose significant health risks to humans.

## 4. Conclusions

A new method based on the QuEChERS extraction protocol and UPLC-MS/MS detection was developed for the analysis of 14 mycotoxins in peaches and their products. The method exhibited high sensitivity as well as excellent linearity, precision, analytical performance, and recovery. The method was successfully applied to the detection of mycotoxins in 109 fresh peaches, 100 diseased peaches, and 89 peach products. The results showed that no mycotoxin was detected in 109 fresh peaches. Six types of mycotoxins (>LOQ) were identified in rotten peaches, with higher quantities and concentrations in rotten parts compared to the rest. In peach juices, peach wines, dried peaches, and canned peaches, eight, nine, nine, and eleven types of mycotoxins were detected, with BEA being the most common. However, the detected mycotoxin concentrations were low, ranging between LOD and LOQ. This suggests that mycotoxin contamination was not a significant issue for peach products. This is the first report on the levels of mycotoxins in peaches and their products in China. The findings of this study will serve as a valuable reference for promoting food safety risk assessment.

## Figures and Tables

**Table 1:** Retention time and MS parameters for the analysis of mycotoxins.

Mycotoxin	Ionization	Retention Time (min)	Parent Ion (*m*/*z*)	Daughter Ion (*m*/*z*)	Cone Voltage (V)
AOH	ESI-	2.29	257	213.0, 215.0	45
AME	ESI-	2.66	271	228.0, 256.0	40
TEN	ES-	1.80	413.0	271.0, 141.0	35
ZEA	ESI-	2.53	317.4	130.9, 175.1	45
AFB1	ESI+	2.08	313	241.2, 285.2	45
AFB2	ESI+	2.01	315.1	259.2, 287.2	45
AFG1	ESI+	1.96	329	200.2, 215.2	45
AFG2	ESI+	1.89	331	189.2, 217.2	45
OTA	ESI+	2.48	404	239.0, 358.0	30
OTB	ESI+	2.32	369.9	103.1, 205.1	25
BEA	ESI+	2.99	801.4	244.2, 262.2	32
ENA	ESI+	3.23	699	228.0, 210.0	28
ENA1	ESI+	3.18	685	210.0, 228.0	32
ENB1	ESI+	3.02	672	100.0, 196.0	29

**Table 2:** Linear regression equation, LOD, LOQ, and matrix effect of 14 mycotoxins in peaches and 4 peach products.

Matrix	Analyte	Linear Regression Equation	Calibration Range (µg/L)	r2	LOD (µg/L)	LOQ (µg/L)	ME
Peach	AOH	y = 7.54796x + 838.429	5–1000	0.995922	0.1	5	−0.9994
	AME	y = 141.154x + 345.321	2–500	0.990956	0.02	5	−0.1183
	TEN	y = 22.2026x + 2829.26	5–500	0.99144	0.2	5	1.5079
	ZEA	y = 20.0209x + 1863.53	5–500	0.99364	0.5	5	−0.1468
	AFB1	y = 122.709x + 12758.3	5–500	0.991648	0.05	5	−0.7668
	AFB2	y = 501.183x + 120.324	2–500	0.99965	0.005	2	−0.3828
	AFG1	y = 384.647x + 36757.6	2–500	0.99304	0.002	5	−0.6386
	AFG2	y = 124.378x + 9920.08	2–500	0.99364	0.05	2	−0.0689
	OTA	y = 9.02856x + 620.06	5–500	0.997128	0.05	5	0.794
	OTB	y = 26.5723x + 2406.69	5–500	0.997947	0.05	5	0.0479
	BEA	y = 3201.13x + 3897.59	2–500	0.995891	0.001	5	−0.0951
	ENA	y = 2113.18x + 2125.74	5–500	0.995857	0.001	5	−0.2242
	ENA1	y = 2288.23x − 616.035	5–500	0.997949	0.001	5	0.5926
	ENB1	y = 1600.89x + 3791.16	2–500	0.991323	0.02	5	0.1839
Peach juice	AOH	y = 11.2376x − 19.2458	2–500	0.991274	0.1	2	0.3577
	AME	y = 120.66x + 626.241	5–500	0.993263	0.005	5	−0.0844
	TEN	y = 14.904x − 28.3899	5–500	0.990278	0.2	5	0.6835
	ZEA	y = 22.4055x + 25.457	2–500	0.998625	0.5	5	−0.486
	AFB1	y = 226.072x − 233.145	5–500	0.990014	0.05	5	−0.5704
	AFB2	y = 559.227x − 790.339	5–500	0.991007	0.02	5	−0.6592
	AFG1	y = 464.872x − 360.002	5–500	0.994151	0.02	5	0.7186
	AFG2	y = 145.942x − 440.789	5–500	0.996035	0.05	5	0.0925
	OTA	y = 2.33123x − 13.2666	5–500	0.995441	0.02	5	−0.5368
	OTB	y = 25.4068x − 118.812	5–500	0.991078	0.5	5	0.0019
	BEA	y = 3901.23x + 7112.98	2–500	0.995076	0.002	2	0.0895
	ENA	y = 1433.96x − 6253.38	5–500	0.995853	0.001	5	−0.1791
	ENA1	y = 2253.03x − 11486.1	5–500	0.99032	0.002	2	0.4337
	ENB1	y = 1409.9x + 2570.79	2–500	0.9928	0.002	5	0.2622
Peach wine	AOH	y = 10.1438x + 13.8803	5–500	0.994728	0.2	5	0.199
	AME	y = 156.222x + 625.792	5–500	0.995025	0.05	5	0.1855
	TEN	y = 13.8082x − 1.33786	5–500	0.990515	0.5	5	0.5597
	ZEA	y = 22.3455x − 24.1925	5–500	0.998802	0.5	5	−0.0478
	AFB1	y = 96.8902x − 342.984	5–500	0.990201	0.05	5	−0.8159
	AFB2	y = 160.054x − 687.431	5–500	0.998139	0.1	5	−0.9025
	AFG1	y = 382.84x − 1062.08	2–500	0.994191	0.05	2	−0.6404
	AFG2	y = 96.164x − 867.755	5–500	0.996312	0.1	5	−0.2801
	OTA	y = 1.83588x − 162.1456	5–500	0.991823	0.1	5	−0.6352
	OTB	y = 26.8597x − 100.638	5–500	0.992604	0.2	5	0.0592
	BEA	y = 3816.12x + 1726.49	2–500	0.990235	0.005	2	0.0657
	ENA	y = 1293.02x − 7563.09	5–500	0.991861	0.005	5	−0.1606
	ENA1	y = 2413.95x − 12259.7	5–500	0.998278	0.005	5	0.2928
	ENB1	y = 1441.67x + 2924.4	2–500	0.990008	0.01	2	0.3524
Dried peach	AOH	y = 9.03485x − 46.9728	5–500	0.997586	0.05	5	0.0679
	AME	y = 79.9475x + 204.936	5–500	0.991232	0.2	5	−0.3933
	TEN	y = 1.48203x − 7.7823	5–500	0.997726	0.5	5	−0.8325
	ZEA	y = 22.5768x − 73.6506	5–500	0.99259	0.2	5	−0.0379
	AFB1	y = 115.606x − 408.308	5–500	0.997732	0.02	5	−0.7803
	AFB2	y = 410.516x − 1613.64	5–500	0.996872	0.01	5	−0.1926
	AFG1	y = 379.021x − 813.538	5–500	0.997744	0.01	5	0.4012
	AFG2	y = 102.06x − 403.805	5–500	0.991837	0.002	5	−0.2359
	OTA	y = 2.44353x − 16.8623	5–500	0.995904	0.1	5	−0.5144
	OTB	y = 24.5706x − 132.813	5–500	0.990434	0.1	5	−0.031
	BEA	y = 2584.51x + 4308.59	5–500	0.995019	0.001	5	−0.2782
	ENA	y = 150.929x − 935.576	5–500	0.993181	0.01	5	−0.1575
	ENA1	y = 459.574x − 2456.14	5–500	0.994547	0.005	5	−0.849
	ENB1	y = 1447.42x + 3642.98	5–500	0.990744	0.005	5	−0.7425
Canned peach	AOH	y = 3.49558x + 0.224697	2–500	0.990619	0.05	2	−0.5777
	AME	y = 150.262x + 0971.747	5–500	0.990368	0.05	5	0.1403
	TEN	y = 13.9001x + 26.7821	5–500	0.990962	0.5	5	0.5701
	ZEA	y = 26.172x − 3.99094	2–500	0.998111	0.2	5	0.1114
	AFB1	y = 150.025x − 631.93	5–500	0.995683	0.02	5	−0.7149
	AFB2	y = 472.453x − 2400.28	5–500	0.994168	0.02	5	−0.7121
	AFG1	y = 479.086x − 1849.07	5–500	0.995939	0.002	5	0.7712
	AFG2	y = 136.814x − 644.595	5–500	0.994183	0.02	5	0.0242
	OTA	y = 2.75636x − 16.9416	5–500	0.992478	0.05	5	−0.4523
	OTB	y = 29.084x − 119.389	5–500	0.990257	0.02	5	0.1469
	BEA	y = 4348.86x + 5791.63	2–500	0.994447	0.002	2	0.2145
	ENA	y = 2021.39x − 5612.6	5–500	0.991561	0.005	5	0.1307
	ENA1	y = 2816.81x − 535.387	2–500	0.999331	0.005	2	1.021
	ENB1	y = 1941.94x + 2519.52	2–500	0.994629	0.01	2	0.52

**Table 3:** Recoveries, intra-day and inter-day RSD for target mycotoxins in different matrices at four spiking levels.

Analyte	Spiking Level (μg/L)	Peach			Peach Juice			Peach Wine			Dried Peach			Canned Peach		
		R ^a^	RSD_r_^b^	RSD_R_^c^	R ^a^	RSD_r_^b^	RSD_R_^c^	R ^a^	RSD_r_^b^	RSD_R_^c^	R ^a^	RSD_r_^b^	RSD_R_^c^	R ^a^	RSD_r_^b^	RSD_R_^c^
AOH	5	93.4	10.7	1.8	92.2	7.1	6.4	92.4	16.0	8.1	104.1	16.1	3.5	98.9	6.9	2.4
	10	101.1	12.0	3.1	101.3	6.3	0.8	110.8	5.2	6.4	104.8	11.7	0.7	100.8	16.7	1.5
	50	100.9	5.7	6.2	92.6	4.7	7.6	97.9	10.9	2.6	93.6	12.2	3.9	94.6	14.2	4.1
	100	87.8	5.6	9.2	103.4	7.9	1.3	88.0	5.4	10.6	104.1	6.6	0.5	100.1	5.9	4.6
AME	5	101.3	5.1	3.9	92.2	10.2	5.1	100.7	7.1	2.3	93.4	4.7	5.6	108.4	6.1	3.8
	10	89.3	5.3	5.3	103.9	6.6	3.1	93.4	3.6	3.3	98.1	5.8	2.7	102.6	4.7	2.4
	50	103.2	3.0	2.5	103.3	12.1	1.9	98.0	2.8	1.2	101.3	3.4	1.5	95.8	2.4	2.5
	100	99.0	1.3	3.0	98.2	3.7	3.7	97.6	2.8	3.9	108.3	9.1	3.5	95.0	4.6	5.8
TEN	5	101.3	10.8	5.7	101.7	8.1	0.8	100.6	12.6	3.4	87.1	14.9	8.9	100.9	10.8	5.9
	10	101.7	11.7	7.5	99.6	11.2	7.1	102.0	15.7	4.3	100.0	14.5	3.2	99.4	6.8	3.4
	50	101.4	9.0	3.2	97.3	10.0	7.5	91.8	7.7	3.1	97.3	10.8	5.0	95.2	11.5	3.9
	100	97.1	6.7	6.1	101.4	8.8	2.9	99.9	11.2	3.1	105.1	11.5	1.5	92.7	11.0	4.0
ZEA	5	96.3	4.1	5.8	95.7	9.6	2.6	102.2	12.0	2.2	99.0	16.1	4.4	108.1	8.1	3.2
	10	93.6	16.1	8.9	98.5	5.6	3.3	98.3	13.9	3.6	99.4	11.9	1.6	102.2	10.6	1.6
	50	104.6	8.7	3.0	109.9	8.9	3.5	89.0	6.2	1.5	104.6	6.6	2.5	89.3	7.1	5.1
	100	92.7	4.6	6.0	96.6	7.4	4.1	96.4	4.5	4.7	101.1	6.1	5.5	100.0	5.1	2.8
AFB1	5	106.2	7.1	5.3	96.4	10.2	3.1	104.8	5.1	0.7	99.9	5.0	3.1	102.5	8.6	6.2
	10	107.2	5.5	4.2	95.6	8.9	7.1	109.4	2.4	6.5	95.3	9.9	6.3	109.6	6.3	6.1
	50	106.1	4.9	9.4	98.9	8.3	10.5	114.4	4.1	5.2	101.8	5.7	1.6	110.3	3.1	19.1
	100	108.3	7.6	10.6	92.8	5.7	5.3	113.2	5.6	7.1	96.2	17.4	4.4	107.8	7.8	6.7
AFB2	5	102.5	9.4	4.3	93.7	10.6	4.7	105.2	8.6	2.3	104.3	4.4	1.1	92.1	6.3	7.3
	10	108.0	6.8	4.0	95.2	11.2	4.6	115.3	3.2	8.3	101.9	8.5	4.3	108.0	8.9	7.6
	50	109.1	5.4	8.3	93.1	7.7	1.9	110.4	8.1	9.4	104.8	7.9	3.9	113.8	5.3	13.9
	100	107.7	5.3	10.9	96.2	11.4	2.5	104.6	7.0	7.9	97.9	11.0	5.4	113.6	6.1	5.1
AFG1	5	104.3	7.3	4.3	98.9	14.5	2.3	108.6	5.7	5.3	107.6	7.5	4.0	106.3	5.8	7.2
	10	103.3	11.6	2.3	101.1	9.8	3.0	105.9	5.4	2.4	103.1	9.4	7.0	111.1	4.3	5.8
	50	101.9	3.6	3.5	101.7	8.0	5.6	109.5	6.7	9.7	101.3	7.1	2.7	109.9	4.9	10.0
	100	104.5	2.3	2.5	95.7	4.7	3.0	108.3	7.0	5.5	99.6	14.3	3.3	106.5	3.9	5.7
AFG2	5	105.9	12.9	1.7	101.2	11.3	3.2	107.9	6.4	5.8	105.9	8.3	1.5	97.8	9.1	3.9
	10	110.1	10.4	4.9	100.8	7.7	3.7	108.1	8.4	3.8	100.1	9.4	3.7	109.2	5.2	6.8
	50	113.4	4.7	5.5	91.3	9.7	7.3	106.8	8.7	8.2	102.8	3.4	3.2	113.5	4.1	11.8
	100	107.1	2.6	7.1	89.2	4.8	7.5	106.5	4.0	4.8	103.5	16.2	2.0	111.1	3.1	9.5
OTA	5	113.2	5.6	11.2	101.7	8.0	3.0	100.6	10.1	1.7	106.0	7.1	4.1	102.9	7.1	1.7
	10	103.1	7.7	0.6	100.0	10.2	2.4	112.0	2.5	10.0	105.4	7.8	5.6	109.7	2.1	11.2
	50	109.8	10.8	8.7	107.6	6.4	5.1	111.0	6.1	8.2	104.5	6.0	4.0	102.7	4.9	11.0
	100	108.0	5.5	10.1	102.2	8.0	6.9	113.0	3.3	8.1	99.2	10.7	2.5	110.4	4.7	4.9
OTB	5	117.6	9.4	16.1	100.0	10.2	1.5	115.4	3.3	8.5	94.9	7.6	3.1	110.9	6.6	8.1
	10	112.6	7.7	6.4	104.0	4.8	4.2	110.8	3.5	5.9	101.5	13.4	3.6	112.9	3.6	14.1
	50	112.7	7.0	10.8	110.0	5.7	8.0	110.6	6.7	6.1	98.1	9.8	4.6	108.6	6.2	1.5
	100	108.9	4.5	7.9	98.2	5.7	0.5	106.9	15.2	12.4	101.3	11.6	5.7	110.1	5.5	4.8
BEA	5	103.6	1.5	0.9	100.2	1.4	1.8	102.1	1.3	1.3	99.5	0.7	0.7	102.0	0.7	2.1
	10	99.0	0.7	1.3	96.1	1.2	5.7	99.1	3.2	1.0	98.4	0.8	2.2	101.7	1.0	2.1
	50	97.5	2.9	4.5	99.5	1.1	1.1	101.3	1.0	1.6	98.9	1.2	2.0	100.9	0.9	0.9
	100	99.9	0.8	0.7	99.3	0.8	1.3	101.2	1.0	2.2	98.9	1.2	0.6	100.5	0.8	1.1
ENA	5	103.7	13.1	5.3	104.4	10.0	13.4	103.3	7.2	5.8	98.1	14.1	3.8	95.3	1.1	5.1
	10	111.7	5.5	2.5	94.8	2.4	5.0	107.7	5.0	2.3	106.5	9.8	6.3	110.9	6.6	4.8
	50	97.5	2.9	3.6	87.6	2.0	12.7	102.5	15.8	5.3	101.7	5.2	0.6	107.9	5.9	5.7
	100	107.7	9.4	0.4	103.8	2.4	3.7	108.9	11.3	2.7	103.0	13.7	1.6	109.1	4.5	3.3
ENA1	5	110.5	6.3	7.8	107.8	6.2	5.3	94.6	1.4	3.5	99.0	5.0	5.0	93.7	1.4	6.0
	10	98.2	3.2	11.0	91.7	9.4	8.8	91.6	6.9	10.0	100.2	4.6	5.0	104.5	5.3	1.8
	50	99.6	6.0	5.2	96.1	1.5	9.1	96.8	2.1	1.5	99.7	5.1	6.1	97.6	2.8	2.6
	100	100.5	2.7	1.7	90.1	3.0	7.0	100.2	0.8	0.5	98.9	10.1	3.7	98.2	2.7	4.3
ENB1	5	91.6	2.9	1.9	108.8	4.9	6.6	97.1	3.0	4.1	96.6	2.5	1.3	100.1	2.9	2.1
	10	96.0	3.6	0.7	84.6	2.7	11.8	97.4	1.2	1.1	98.2	3.5	3.7	109.0	6.4	3.0
	50	100.1	2.7	2.0	88.7	4.0	9.1	99.2	3.4	3.6	100.5	2.9	3.2	94.6	3.9	0.9
	100	96.2	2.4	4.3	97.8	2.5	4.2	99.2	2.8	2.6	101.2	10.8	1.8	102.0	1.4	1.2

^a^ Recoveries (*n* = 6, %); ^b^ intra-day (*n* = 6, %); ^c^ inter-day (*n* = 3, %).

**Table 4:** Contamination levels of mycotoxins in diseased peaches.

Mycotoxin	Part	Positive Sample	Frequency (%)	Range (µg/kg)	Mean (µg/kg)	Median (µg/kg)
AOH	A	61	31.0	5.2–1664.3	207.9	28.3
	B	50	25.4	8.9–27.1	15.3	9.7
AME	A	65	33.0	5.3–1024.5	102.9	16.6
	B	59	29.9	<LOQ	NQ	NQ
TEN	A	32	16.2	5.1–139.8	32.9	11.4
	B	25	12.7	<LOQ	NQ	NQ
ZEA	A	12	6.1	<LOQ	NQ	NQ
	B	8	4.1	<LOQ	NQ	NQ
AFB1	A	20	10.2	41.9	41.9	41.9
	B	11	5.6	<LOQ	NQ	NQ
AFB2	A	72	36.5	<LOQ	NQ	NQ
	B	72	36.5	<LOQ	NQ	NQ
AFG1	A	94	47.7	<LOQ	NQ	NQ
	B	97	49.2	<LOQ	NQ	NQ
AFG2	A	50	25.4	<LOQ	NQ	NQ
	B	50	25.4	<LOQ	NQ	NQ
OTA	A	57	28.9	<LOQ	NQ	NQ
	B	41	20.8	<LOQ	NQ	NQ
OTB	A	28	14.2	<LOQ	NQ	NQ
	B	20	10.2	<LOQ	NQ	NQ
BEA	A	97	49.2	7.0–127.7	49.5	13.7
	B	100	50.8	5.2	5.2	5.2
ENA	A	97	49.2	<LOQ	NQ	NQ
	B	100	50.8	<LOQ	NQ	NQ
ENA1	A	96	48.7	<LOQ	NQ	NQ
	B	100	50.8	<LOQ	NQ	NQ
ENB1	A	29	14.7	<LOQ	NQ	NQ
	B	20	10.2	<LOQ	NQ	NQ

ND, not detected; NQ, not quantifiable.

**Table 5:** Contamination levels of mycotoxins in peach products.

Mycotoxin	Positive Sample (*n*)/Frequency (%)			
	Peach Juice	Peach Wine	Dried Peach	Canned Peach
AOH	ND	ND	1 (5.6)	4 (13.3)
AME	1 (7.1)	7 (25.0)	1 (5.6)	6 (20)
TEN	1 (7.1)	ND	ND	ND
ZEA	ND	ND	ND	ND
AFB1	ND	4 (14.3)	2 (11.1)	4 (13.3)
AFB2	ND	6 (21.4)	6 (33.3)	2 (6.7)
AFG1	ND	ND	ND	8 (26.7)
AFG2	1 (7.1)	5 (17.9)	8 (44.4)	3 (10.0)
OTA	2 (14.3)	ND	ND	ND
OTB	ND	1(3.6)	ND	8 (26.7)
BEA	14 (100.0)	28 (100.0)	18 (100.0)	22 (73.3)
ENA	12 (85.7)	26 (92.9)	9 (50.0)	14 (46.7)
ENA1	14 (100.0)	28 (100.0)	17 (94.4)	20 (66.7)
ENB1	14 (100.0)	27 (96.4)	17 (94.4)	16 (53.3)

ND, not detected.

## Data Availability

Data are contained within the article.

## Supplementary Materials

The following supporting information can be downloaded at: https://www.mdpi.com/article/10.3390/foods12173216/s1, Figure S1: Geographic location of peach samples collected from China; Figure S2: The fresh and deceased peach samples; Figure S3: UPLC-MS/MS chromatogram of a mixture of mycotoxin standards (100 μg/mL) obtained in MRM mode; Table S1: Peach sampling locations in China; Table S2: The geographic distribution of diseased and fresh peaches in China.
