# Supplementary material for: Mycotoxin Determination in Peaches and Peach Products with a Modified QuEChERS Extraction Procedure Coupled with UPLC-MS/MS Analysis
Source: Foods. 2023 Aug 26;12(17):3216. doi: 10.3390/foods12173216 (PMC10487233; doi:10.3390/foods12173216)
Supplement: Supplementary file 1 [file foods-12-03216-s001.zip › foods-2551110-supplementary.pdf]

Table S1. Peach sampling locations in China.

No.	Location	Longitude	Latitude
1	Beixiang Village, Beixiang Town, Yuxian County, Yanhu District, Yuncheng City, Shanxi Province	110.9343	35.1443
2	lujiazhuang, Guoxiang Road, Yanhu District, Yuncheng City, Shanxi Province.	111.1727	35.3060
3	Yangli Village, Hanxue Town, Wanrong County, Yuncheng City, Shanxi Province	110.8678	35.3049
4	Beizhang Village, Daqu Town, Yanhu District, Yuncheng City, Shanxi Province	110.9237	35.0489
5	Beigaoyu Village, Jiezhou Town, Yanhu District, Yuncheng City, Shanxi Province	110.8312	34.9630
6	Jinjing Town, Yanhu District, Yuncheng City, Shanxi Province	110.7927	34.9915
7	Peijie Village, Peijie Town, Xia County, Yuncheng City, Shanxi Province	111.1162	35.1170
8	Xialiu Village, Yaofeng Town, Xia County, Yuncheng City, Shanxi Province	111.1768	35.1543
9	Qiwu Village, Huzhang Town, Xia County, Yuncheng City, Shanxi Province.	111.2181	35.2220
10	Xingnan Village, Shuitou Town, Xia County, Yuncheng City, Shanxi Province	111.1097	35.1930
11	Pantao Yuan Family Farm, Jianlingjiao Village, Yuandong Town, Jindong District, Jinhua City, Zhejiang Province	119.7937	29.2680
12	Lüxi County, Honghe Prefecture, Yunnan Province	103.7759	24.4892
13	Xinrong Ecological Agriculture Development Co., Ltd., Baxianxiaqishan Ecological Farm, Gutian County, Ningde City, Fujian Province	118.8490	26.5655
14	Xinrong Ecological Agriculture Development Co., Ltd., Baxianxiaqishan Ecological Farm, Gutian County, Ningde City, Fujian Province	118.8488	26.5643
15	Xinrong Ecological Agriculture Development Co., Ltd., Baxianxiaqishan Ecological Farm, Gutian County, Ningde City, Fujian Province	118.8486	26.5660
16	Yangdu Base, No. 11 Chenjia Stone Bridge, Yangdu Village, Haining City, Zhejiang Academy of Agricultural Sciences, Zhejiang Province	120.4199	30.4362
17	Qianruiying Village, Pinggu District, Beijing City	117.0496	40.1164
18	Longyaozhuang Village, Huoxianzhen, Tongzhou District, Beijing	116.8520	39.7035
19	Yangdu Base, No. 11 Chenjia Stone Bridge, Yangdu Village, Haining City, Zhejiang Academy of Agricultural Sciences, Zhejiang Province	120.4178	30.4366

20	Urban Peach Blossom Land, No. 8-26, Changshan Street, Feicheng, Tai'an City, Shandong Province	116.7799	36.1779
21	Tianpinghu Road, Daiyue District, Tai'an City, Shandong Province	117.0290	36.2179
22	Xili Village, Feicheng City, Shandong Province	116.6692	36.1455
23	Xili Village, Feicheng City, Shandong Province	116.6714	36.1433
24	Yejiadayuan,Longquanyiqu,Chengdu City,Sichuan Province	104.3378	30.5495
25	Yejiadayuan,Longquanyiqu,Chengdu City,Sichuan Province	104.3381	30.5522
26	Longquanshengtaiguoyuan,Longquanyiqu,Chengdu City,Sichuan Province	104.2865	30.5298
27	Longquanshengtaiguoyuan,Longquanyiqu,Chengdu City,Sichuan Province	104.2872	30.5294
28-	Shiqiao Brigade, Shiqiao Town, Rencheng District, Jining City, Shandong Province	116.7120	35.3166
29	319, Shuixi Zhanjian Building Materials Market, Jianou City, Nanping City, Fujian Province	118.3165	27.0284
30	Gaozhuang Hu, Dongcheng District, Shan County, Heze City, Shandong Province	116.1679	34.7613
31	Gaozhuang Hu, Dongcheng District, Shan County, Heze City, Shandong Province	116.1679	34.7613
32	Caodian Village, Zhongxing Town, Shan County, Heze City, Shandong Province	116.3220	34.7511
33	Caodian Village, Zhongxing Town, Shan County, Heze City, Shandong Province	116.3321	34.7466
34	Haizhuang Village, Changli County, Hebei Province	119.5250	39.9807
35	Haizhuang Village, Changli County, Hebei Province	119.0975	39.7027
36	Xueyan Shuimitao Zhongzhijidi， Changzhou City, Jiangsu Province	120.0376	31.5696
37	Taian Station Tianpinghu Demonstration Base, Daiyue District, Taian City, Shandong Province	117.0244	36.2187
38	Taian Station Tianpinghu Demonstration Base, Daiyue District, Taian City, Shandong Province	117.0255	36.2176
39	Taian Station Tianpinghu Demonstration Base, Daiyue District, Taian City, Shandong Province	117.0245	36.2164
40	Taian Station Tianpinghu Demonstration Base, Daiyue District, Taian City, Shandong Province	117.0249	36.2151
41	Heishishan, Gaodu Town, Mengyin County, Linyi City, Shandong Province	117.9509	35.8646

42	Gangjiagou Village, Gaodu Town, Mengyin County, Linyi City, Shandong Province	117.9520	35.8199
43	Xia'aigu Village,Bancheng town, Lanshan District, Linyi City, Shandong Province, China	118.3661	35.2656
44	Wu Liyang Village, Zhuangmiao Town, Cao County, Heze City, Shandong Province, China	115.4099	34.9465
45	Jinzhu Fruit Forest Professional Cooperative, Haodian Town, Guangshui City, Suizhou City, Hubei Province, China	113.7473	31.7723
46	Nancaopo Village, Gaoxing Town, Lanshan District, Rizhao City, Shandong Province, China	119.3717	35.3322
47	Hongweicun, Jiaolaizhen, Jiaozhoushi,Qingdao city, Shandong province	120.0273	36.9309
48	Bei Village, Dazhangzhuang Town, Yiyuan County, Zibo City, Shandong Province	118.0287	36.0393
49	Caojiazhuang Village, Dazhangzhuang Town, Yiyuan County, Zibo City, Shandong Province.	118.0904	36.0668
50	Lianggecun,Qizhen,Mei County, Baoji City, Shaanxi Province	107.7416	34.2896
51	Xizhai Village, Huaiya Town, Mei County, Baoji City, Shaanxi Province	107.9995	34.1286
52	Xizhai Village, Huaiya Town, Mei County, Baoji City, Shaanxi Province	107.9966	34.1306
53	Houshizi Village, Guanzhuang Town, Anqiu City, Weifang City, Shandong Province	119.1451	36.2390
54	Houshizi Village, Guanzhuang Town, Anqiu City, Weifang City, Shandong Province	119.1435	36.2398
55	Sanxing Village, Fengqiao Town, Jiaxing City, Zhejiang Province. (Jiahu Jing)	120.8318	30.6728
56	Sanxing Village, Fengqiao Town, Jiaxing City, Zhejiang Province. (Jiahu Jing)	120.8303	30.6712
57	Dongnangtong Village, Liyuan Street Office, Pingdu City, Qingdao city, Shandong Province	119.8643	36.7935
58	Dianzi Town, Pingdu City, Qingdao city, Shandong Province	119.9352	36.8995
59	Chenjia Hetou Village, Jiaobei Town, Jiaozhou City, Shandong Province	119.9836	36.3783
60	Fujia Village, Jiaozhou City, Shandong Province	119.9625	36.2526
61	Changzhi Village, Daxin Town, Jimo District, Qingdao City, Shandong Province	120.3829	36.4818
62	Qianjin Village, Daxin Town, Jimo District, Qingdao City, Shandong Province	120.3710	36.4877
63	Qianguang Village, Zhujiachang Town, Yuping County, Guiyang City, Guizhou Province	108.9818	27.3665

64	Zhujiachang Town, Yuping County, Guiyang City, Guizhou Province	108.9818	27.3665
65	Kongzhuang Village, Changli County, Qinhuangdao City, Hebei Province	119.0972	39.6967
66	Haizhuang Village, Changli County, Qinhuangdao City, Hebei Province	119.5256	39.9802
67	Qianxiazhuang Village, Liutian Gezhuang Town, Lulong County, Qinhuangdao City, Hebei Province	118.9894	39.7935
68	Qianxiazhuang Village, Liutian Gezhuang Town, Lulong County, Qinhuangdao City, Hebei Province	118.9867	39.7904
69	Houxiazhuang Village, Liutian Gezhuang Town, Lulong County, Qinhuangdao City, Hebei Province	118.9914	39.7954
70	Lufengkou Village, Funing Town, Funing District, Qinhuangdao City, Hebei Province	119.1610	39.9027
71	Lufengkou Village, Funing Town, Funing District, Qinhuangdao City, Hebei Province	119.1641	39.9037
72	Lufengkou Village, Funing Town, Funing District, Qinhuangdao City, Hebei Province	119.1649	39.9018
73	Houliangshan Village, Houlingshan Township, Changli County, Qinhuangdao City, Hebei Province	119.1951	39.7509
74	Houliangshan Village, Houlingshan Township, Changli County, Qinhuangdao City, Hebei Province	119.1945	39.7493
75	Houliangshan Village, Houlingshan Township, Changli County, Qinhuangdao City, Hebei Province	119.2074	39.7462
76	Dasijiazhuang Village, Donganzhuang Town, Shenzhou City, Hebei Province	115.5123	37.9649
77	Ximazhuang Village, Mucun Town, Shenzhou City, Hebei Province	115.5057	38.0272
78	Shibei Village, Qianying Township, Xinji City, Hebei Province	115.4428	37.9598
79	Shibei Village, Qianying Township, Xinji City, Hebei Province	115.4431	37.9615
80	Yujiazhuang, Qianying Town, Xinji City, Hebei Province	115.4458	37.9740
81	Dulin Village, Qianying Town, Xinji City, Hebei Province	115.4282	37.9742
82	Renyi Village, Donglizhuang Township, Jinzhou City, Hebei Province	115.0424	37.9965
83	Renyi Village, Donglizhuang Township, Jinzhou City, Hebei Province	115.0421	37.9969
84	Hejiazhai Village, Jinzhou City, Hebei Province	115.0139	37.8951
85	Shangyang Village, Chonglu Township, Jianyang District, Nanping City, Fujian Province	118.2184	27.5319

86	Beiyaluzui Village, Tieshan Town, Lushunkou District, Dalian City, Liaoning Province	121.1836	38.8009
87	Li Tun, Xiaoxutun Village, Xingtai Town, Pulandian District, Dalian City, Liaoning Province	122.4436	39.6013
88	Li Tun, Xiaoxutun Village, Xingtai Town, Pulandian District, Dalian City, Liaoning Province	122.4435	39.6018
88	Nanshan Fruit Orchard, Bali Village, Fuzhou Town, Wafangdian City, Dalian, Liaoning Province	121.7661	39.7620
90	Bali Village, Fuzhou Town, Wafangdian City, Liaoning Province	121.7659	39.7612
91	Bali Village, Fuzhou Town, Wafangdian City, Liaoning Province	121.7677	39.7612
92	Shuanglong Village, Fuzhou Town, Wafangdian City, Liaoning Province, Dalian City	121.7204	39.7748
93	Pinggu District, Beijing City	117.1188	40.1915
94	Pinggu District, Beijing City	117.0143	40.2616
95	Pinggu District, Beijing City	117.1078	40.1856
96	Pinggu District, Beijing City	117.1417	40.1443
97	Pinggu District, Beijing City	117.1152	40.1839
98	Pinggu District, Beijing City	117.1210	40.1917
99	Pinggu District, Beijing City	117.1103	40.1894
100	Pinggu District, Beijing City	117.1181	40.1929
101	Pinggu District, Beijing City	117.1130	40.1903
102	Bailiao Zhou Village, Rulin Town, Chengbu Miao Autonomous County, Shaoyang City, Hunan Province	110.3086	26.3433
103	Yinzi Village, Longmen Town, Pingjiang County, Yueyang City, Hunan Province	114.0425	28.8669
104	Sanjia Village, Maozhu Town, Qiyang County, Yongzhou City, Hunan Province	111.7729	26.5600
105	Bengkan Village, Huanghua Town, Changsha County, Hunan Province	113.1825	28.3131
106	Honey Fruit Peach Professional Cooperative, Xikou Town, Fenghua City, Zhejiang Province	121.2456	29.6689
107	Taiping Lake, Taian Station, Daiyue District, Tai'an City, Shandong Province	117.0247	36.2187

108	Xiangmuqiaocun, Qujiangzhen, Jianshui County, Honghe Prefecture, Yunnan Province	102.8154	23.6010
109	Beiyahu Village, Tie Shan Town, Lushunkou District, Dalian, Liaoning Province	121.1768	38.7858

Table S2. The geographic distribution of diseased and fresh peaches in China.

Location	Diseased peach sample	Fresh peach sample
Beijing city	10	11
Fujian province	4	5
Guizhou province	4	2
Hebei province	19	22
Hubei province	2	1
Hunan province	4	4
Jiangsu province	2	1
Liaoning province	2	8
Shandong province	31	30
Shanxi province	10	10
Shaanxi province	5	3
Sichuan province	1	4
Yunnan province	1	2
Zhejiang province	5	6

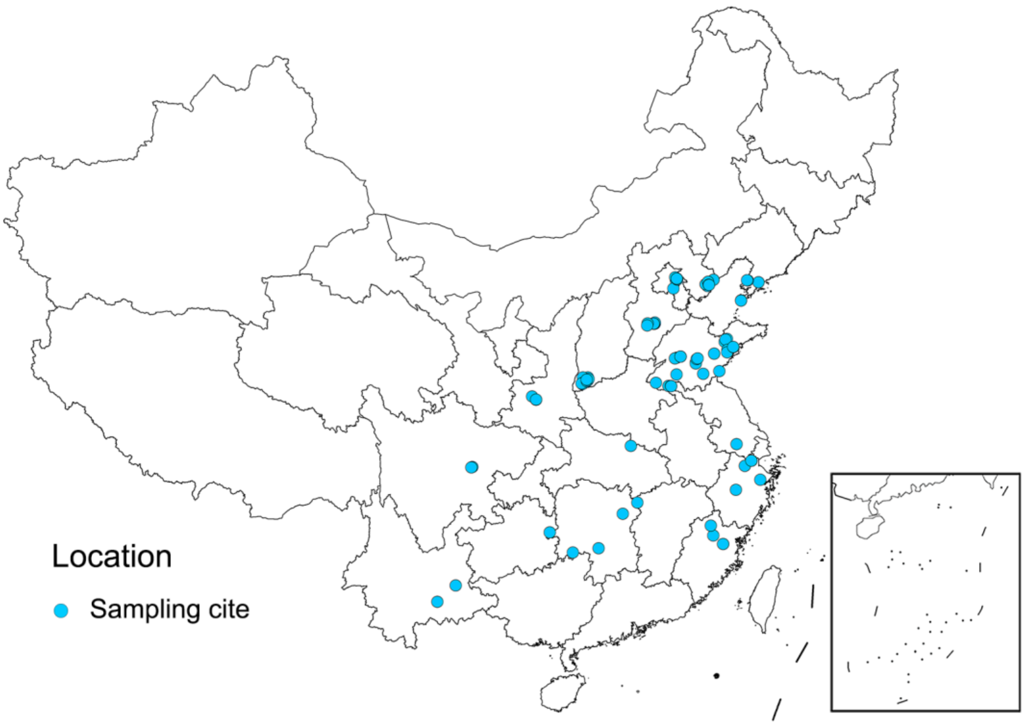

Figure S1. Geographic location of peach samples collected from China

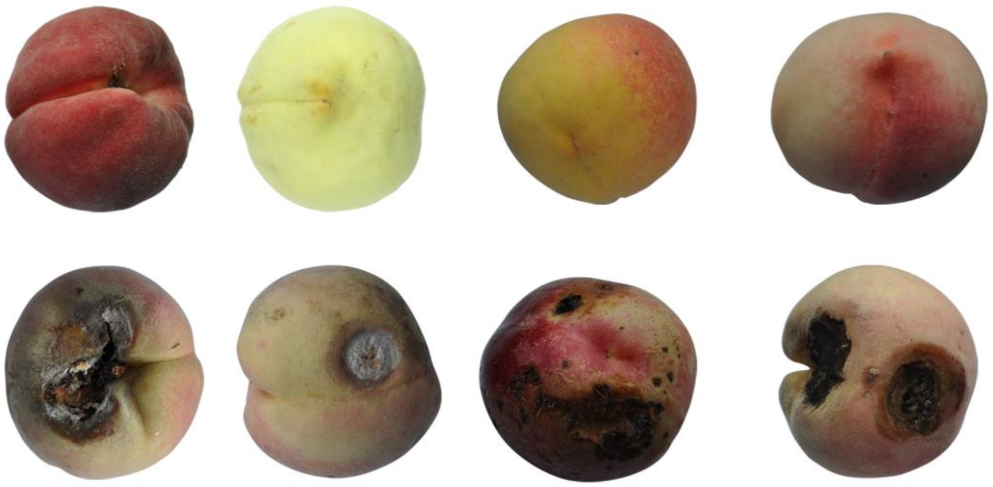

Figure S2. The fresh and diseased peach samples

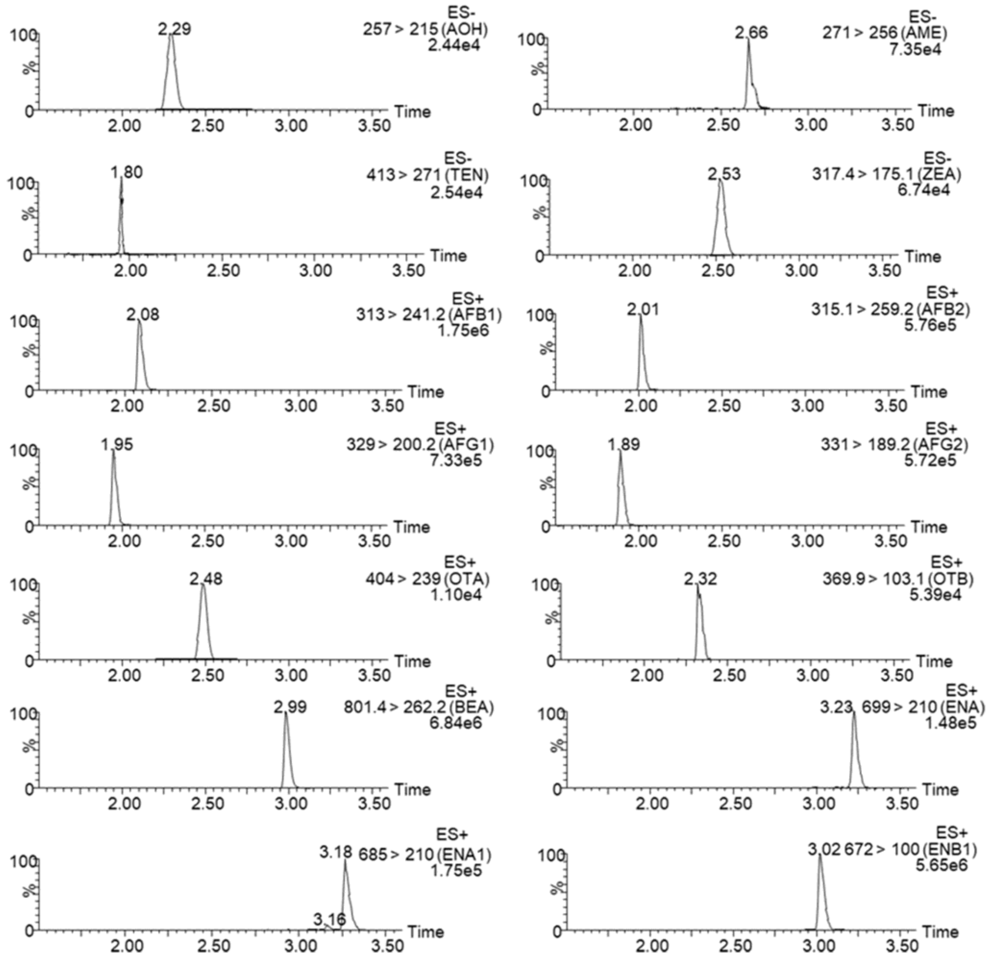

Figure S3. UPLC-MS/MS chromatogram of a mixture of mycotoxin standards (100 μg/mL) obtained in MRM mode.
